# Supplementary material for: Genetic Characterization of Human Rabies Vaccine Strain in Japan and Rabies Viruses Related to Vaccine Development from 1940s to 1980s
Source: Viruses. 2022 Sep 29;14(10):2152. doi: 10.3390/v14102152 (PMC9607234; doi:10.3390/v14102152)
Supplement: Supplementary file 1 [file viruses-14-02152-s001.zip › viruses-1918945-SI-done.pdf]

viruses

MDPI

Article

Genetic Characterization of Human Rabies Vaccine Strain in Japan and Rabies Viruses Related to Vaccine Development from 1940s to 1980s

Madoka Horiya [1,2], Guillermo Posadas-Herrera [1], Mutsuyo Takayama-Ito [1], Yukie Yamaguchi [1], Itoe Iizuka-Shiota [1], Hirofumi Kato [1], Aikou Okamoto [2], Masayuki Saijo [1] and Chang-Kweng Lim [1,*]

[1] Department of Virology I, National Institute of Infectious Diseases, 1-23-1 Toyama, Shinjuku, Tokyo 162-8640, Japan
[2] Department of Obstetrics and Gynecology, The Jikei University School of Medicine, 3-25-8 Nishi-Shinbashi, Minato-ku, Tokyo 105-8461, Japan
* Correspondence: ck@niid.go.jp; Tel.: +81-3-5285-1111; Fax: +81-3-5285-1188

Supplementary Table S1. List of rabies strains.

Strain	Accession No.	Country/Area	Species	Year
DRV-AH08	HQ450385	China	dog	2008
CYN1009D	JQ730682	China	dog	2010
WH11	JQ647510	China	donkey	2011
HN10	EU643590	China: Hunan	human	2006
GX4	GU358653	China: Guangxi	Dog	1994
SH06	GU345748	China: Shanghai	Dog	2006
F04	FJ712196	China	Chinese ferret badger	2008
aG	GQ412744	China: Beijing	Dog	1931
IMDRV-13	KJ564280	Inner Mongolia	Dama dama	2013
8743THA	EU293121	Thailand	Human	1983
R2012-26	JF620487	Taiwan	Ferret badger	2012
08F40	KC171643	South Korea	Raccoon dog	2008
NNV-RAB-H	EF437215	India	Human	unknown
Lao4	AB981664	Laos	Dog	2011
Pk24	HE802676	Pakistan	Mus musculus	2007
H-1413-09	AB635373	Sri Lanka	Paradoxurus zeylonensis	2009
1410KOM	JQ944707	Russia	Deer	2008
Rus(Astrakhan)8330H	KT728349	Russia(Astrakhan)	Homo sapience	2003
9147FRA	EU293115	France	Fox	1991

RV437	KF154997	Estonia	Raccoon dog	un-known
148/lib01041	LN879481	Germany	Vulpes vulpes	1998
RV2516	KF155000	Iraq	Cow	2010
RV2324	KF154998	Israel	Dog	1950
RRV ON-99-2	EU311738	Canada	Raccoon dog	1999
13NO643AFX	KU198478	Canada	Alopex lagopus	2013
1088	AB645847	USA	Marmota monax	un-known
SM5075	JQ685949	USA	Skunk	2001
A11-5300	KC737850	USA	Homo sapience	2011
CASK2	JQ685970	USA	Striped skunk	1974
DRV-Mexico	HQ450386	Mexico	Dog	un-known
3634DR	JQ685936	Mexico	Bovine	2009
MEXSK3636	JQ685975	Mexico	Spilogale putorius	2009
9704ARG	EU293116	Argentina	Tadarida brasiliensis	1997
IP 4005/12	KM594043	Brazil	Bos taurus	2012
BRmk1358	AB810256	Brazil	Cebus apella	un-known
AT6	KU523255	French Guiana	Desmodus rotundus	2010
9001FRA	EU293113	Guyana	Dog (bitten by a bat)	1990
RV2985	KP723638	Ethiopia	Canis simensis	2014
RV2627	KF155001	Morocco	Cow	2009
240K09	JX473841	Namibia	Tragelaphus strepsiceros	2009
DRV-NG11	KC196743	Nigeria	Dog	2011
RV3123.1	KR906792	Tanzania	Domestic dog	2012
21467	KT336435	Zimbabwe	Canis lupus jaminiaris	1993
Komatsugawa	LC553558	Japan	Dog	1940's
14016BOT	KX148218	Botswana	Wild Cat	2009
14018AFS	KX148223	South Africa	Feline	2000
15001AFS	KX148220	South Africa	Mongoose	2013
15003AFS	KX148221	Southe Africa	Squirrel	2014
86097BEN	KX148107	Benin	Cat	1986
86036HAV	KX148234	Burkina Faso	Mouse	1986
97002IND	KX148246	India	Homo sapiens	1997

RV2417	KF154999	United Kingdom	Dog	2008
Yokohama/human	LC628899	Japan from the Philippines	Human	2006
Kyoto	LC571945	Japan from the Philippines	Human	2006
SAD 2006 from Austria	EU886636	Austria	Red fox taking SAD vaccine	2006
4aGV	JN234411	China	vaccine strain	
CTN^1	FJ959397	China	human	1956
ERA-VC	FJ913470	China	vaccine strain	
EPHVAC	JQ944709	Ethiopia	vaccine strain	
CTN-1-31	HQ317918	China	human	1956
ERA	EF206707	Germany	vaccine strain	
SAD Bern Lysbulpen	EF206708	Czech Republic	vaccine strain	
SAG 2	EF206719	France	vaccine strain	
RV-97	EF542830	Russia	vaccine strain	
RB/E3-15	EU182346	USA	vaccine strain	
Flury-LEP	GU565703	China	vaccine strain	
Flury-HEP	GU565704	China	vaccine strain	
CVS-11	GQ918139	China	challenge strain	
CVS N2c	HM535790	China	suckling-mouse-brain-adapted CVS-24	
Ni-CE	AB128149	Japan	Chicken embryo fibroblast passed Nishigahara strain	
PV	NC001542	France	vaccine strain	
PV-2061	JX276550	China	vaccine strain	
ERA	AB781935	South Korea	vaccine strain	
CTNCEC25	KJ466147	China	vaccine strain	
Moscow 3253	KM198893	Russia	vaccine strain	
Flury LEP	DQ099524	Germany	vaccine strain	
PM1503	DQ099525	Germany	vaccine strain	
Nishigahara	AB044824	Japan	vaccine strain	
RC-HL	AB009663	Japan	vaccine strain	

	−14	−10	−9	−8	−6	−4	−3	10	19	# 37	# 40	114	120	126	133	140	147	156	158	164	167
HEP-Flury GU565704	V	A	P	L	V	S	L	K	L	N	G	N	H	K	V	T	K	G	N	V	T
HEP-Flury NIID LC717409	*	*	*	*	*	P	*	*	*	*	*	*	*	*	*	*	*	*	*	*	*
HFOP LC717410	*	*	*	*	*	P	*	*	*	*	*	*	*	*	*	*	*	*	*	*	*
CEF-S LC717412	*	*	*	*	*	P	*	*	*	*	R	*	N	*	*	*	*	*	*	E	*
CEF-L LC717411	*	*	*	*	*	P	*	*	*	*	*	*	*	*	*	*	*	*	*	E	*
LEP Flury GU565703	*	V	*	*	G	*	*	*	I	*	E	*	*	*	*	*	*	*	*	*	*
LEP Flury NIID LC717413	*	V	*	*	*	*	*	*	I	*	*	*	*	*	*	*	*	*	*	*	*
FOP LC717414	*	V	*	*	*	*	*	*	I	*	E	*	*	*	*	*	*	*	*	*	*
Nishigahara AB044824	A	V	*	I	G	*	S	T	I	*	*	S	*	*	*	A	N	S	K	*	V
Nishigahara NIID LC717415	A	V	*	I	G	*	*	T	I	*	*	S	*	*	*	A	N	S	K	*	V
NOPM LC717416	A	V	*	I	G	*	*	T	I	*	E	S	*	*	*	A	N	S	K	*	V
Takamen LC717419	A	V	*	I	G	*	*	T	I	D	*	S	*	*	*	A	N	S	K	*	V
CVS-11 GQ918139	*	V	L	*	G	*	*	E	I	*	E	*	*	R	I	*	*	*	K	*	*
CVS-54 LC717417	*	V	L	*	G	*	*	E	I	*	E	*	*	R	I	*	*	*	K	*	*
COP LC717418	*	V	L	*	G	*	*	E	I	*	E	*	*	R	I	*	*	*	K	*	*
CVS-N2c HM535790	*	V	L	*	G	*	*	E	I	*	E	*	*	R	I	*	*	*	K	*	*

	177	182	183	185	186	188	194	202	204	206	224	236	247	255	259	263	273	291	303	326	! 333	346	349	367	369	385	389	403	407	412	416
HEP-Flury GU565704	I	N	L	L	G	S	H	K	D	T	K	M	D	G	N	F	V	M	H	D	Q	R	E	S	G	M	E	S	D	V	V
HEP-Flury NIID LC717409	*	*	*	*	*	*	*	*	*	*	*	*	*	*	*	*	*	*	*	*	*	*	*	*	*	*	*	*	*	*	*
HFOP LC717410	*	*	*	*	*	*	*	*	*	*	I	*	*	*	*	*	*	*	*	*	*	*	*	*	*	*	*	*	*	*	*
CEF-S LC717412	*	*	*	*	*	*	*	*	*	*	I	*	V	*	*	D	*	*	*	*	*	*	*	*	*	*	*	*	*	*	*
CEF-L LC717411	*	*	*	*	*	*	*	*	*	*	I	*	V	*	*	D	*	*	*	*	*	*	*	*	*	*	*	*	*	*	*
LEP Flury GU565703	*	*	*	*	*	*	N	*	G	*	*	*	*	*	*	*	*	*	*	*	R	*	G	*	*	*	*	*	*	*	*
LEP Flury NIID LC717413	*	*	*	*	*	*	N	*	G	*	*	*	*	*	*	*	*	*	*	*	R	*	G	*	*	*	*	*	*	*	*
FOP LC717414	*	*	*	*	*	*	N	*	G	*	*	*	*	*	*	*	*	*	*	*	R	*	G	*	*	*	*	*	*	G	*
Nishigahara AB044824	V	S	*	*	*	*	N	*	S	*	*	*	N	D	*	D	I	I	Y	A	R	*	G	P	*	I	*	R	*	T	I
Nishigahara NIID LC717415	V	S	*	*	*	*	N	*	S	*	*	*	N	D	*	D	I	I	*	A	R	*	G	P	*	I	*	R	*	T	I
NOPM LC717416	V	S	*	*	*	*	K	*	S	*	*	*	N	D	*	D	I	I	*	A	R	*	G	P	*	I	*	R	*	T	I
Takamen LC717419	V	S	*	*	*	*	N	*	S	*	*	*	N	D	*	D	I	I	*	A	R	*	G	P	*	I	*	R	*	T	I
CVS-11 GQ918139	*	*	P	P	R	P	N	N	N	*	R	*	*	D	*	*	*	*	*	*	R	K	G	P	D	*	K	*	E	A	*
CVS-54 LC717417	*	*	P	P	R	P	N	N	N	*	R	*	*	D	*	*	*	*	*	*	R	K	G	P	D	*	*	*	E	A	*
COP LC717418	*	*	P	P	R	P	N	N	N	*	R	*	*	D	*	*	*	*	*	*	R	K	G	P	D	*	*	*	E	A	*
CVS-N2c HM535790	*	*	P	P	R	P	N	N	N	*	R	*	*	D	*	*	*	*	*	*	R	K	G	P	D	*	*	*	E	A	*

	424	425	427	431	436	439	443	444	447	448	450	452	455	456	460	463	464	465	469	470	472	473	474	475	476	479	490	492	498	503	504
HEP-Flury GU565704	H	K	V	D	K	K	M	I	A	L	A	M	I	F	C	R	V	N	S	T	S	N	L	G	G	R	V	S	K	T	R
HEP-Flury NIID LC717409	*	*	*	*	*	*	*	*	*	*	*	*	*	*	*	*	*	*	*	*	*	*	*	*	*	*	*	*	*	*	*
HFOP LC717410	*	*	*	*	*	*	*	*	*	*	*	*	*	*	*	*	*	*	*	*	*	*	*	*	*	*	*	*	*	*	I
CEF-S LC717412	*	*	*	*	*	*	*	*	*	*	*	*	*	*	*	*	*	*	*	*	*	*	*	*	*	*	*	*	*	*	I
CEF-L LC717411	*	*	*	*	*	*	*	*	*	*	*	*	*	*	*	*	*	*	*	*	*	*	*	*	*	*	*	*	*	*	I
LEP Flury GU565703	*	*	*	E	N	*	*	*	*	*	*	*	*	*	*	*	*	*	*	*	*	S	*	*	E	*	*	*	*	*	R
LEP Flury NIID LC717413	*	*	*	*	N	*	*	*	*	*	*	*	*	*	*	*	*	*	*	*	*	S	*	*	*	*	*	*	*	*	R
FOP LC717414	*	*	*	*	N	*	*	*	*	*	*	*	*	*	*	*	*	*	*	*	*	S	*	*	*	*	*	*	*	*	R
Nishigahara AB044824	*	E	*	*	N	E	L	S	T	*	*	*	*	*	*	K	*	D	*	*	R	S	*	R	*	*	F	P	*	*	G
Nishigahara NIID LC717415	*	E	*	*	N	E	L	S	T	*	*	*	*	*	*	K	*	D	*	*	R	S	*	R	*	*	F	P	*	*	G
NOPM LC717416	*	E	*	*	N	E	L	S	T	*	*	*	*	*	*	K	*	D	*	*	R	S	*	R	*	*	F	P	*	*	G
Takamen LC717419	*	E	*	*	N	E	L	S	T	*	*	*	*	*	*	K	*	D	*	*	R	S	*	R	*	*	F	P	*	*	G
CVS-11 GQ918139	Y	*	I	*	N	*	*	T	*	M	G	V	F	S	W	*	A	*	*	K	R	S	F	*	*	G	*	D	R	I	*
CVS-54 LC717417	Y	*	I	*	N	*	*	T	*	M	G	V	F	S	W	*	A	*	*	K	R	S	F	*	*	G	*	D	*	I	*
COP LC717418	Y	*	I	*	N	*	*	T	*	M	G	V	F	S	W	*	A	*	L	K	R	S	F	*	*	G	*	D	*	I	*
CVS-N2c HM535790	Y	*	I	*	N	*	*	T	*	M	G	V	F	S	W	*	A	*	*	K	R	S	F	*	*	G	*	D	*	I	*

Supplementary Figure S1. Comparison of the deduced amino acid sequences of rabies virus G protein. Amino acid sequence deduced from the nucleotide sequence of rabies viruses which were investigated in our study are depicted with one-letter symbols and aligned together with those of five other strains, HEP-Flury (GU565704), LEP-Flury (GU565703), Nishigahara (AB044824), CVS-11 (GQ918139), and CVS-N2c (HM535790). In this representation, only the sequence of the HEP-Flury (GU565704) strain is depicted when amino acid residues, at each corresponding position,

were identical among the 15 strains. Antigenic sites II are marked with "#". Position 333 was marked with "!".
